# Wider geographic distribution and higher diversity of hexaploids than tetraploids in *Carassius* species complex reveal recurrent polyploidy effects on adaptive evolution

**DOI:** 10.1038/s41598-017-05731-0

**Published:** 2017-07-14

**Authors:** Xiao-Li Liu, Fang-Fang Jiang, Zhong-Wei Wang, Xi-Yin Li, Zhi Li, Xiao-Juan Zhang, Fan Chen, Jian-Feng Mao, Li Zhou, Jian-Fang Gui

**Affiliations:** State Key Laboratory of Freshwater Ecology and Biotechnology, Institute of Hydrobiology, Chinese Academy of Sciences, University of the Chinese Academy of Sciences, Wuhan, 430072 Hubei China

## Abstract

Polyploidy roles on adaptive evolution and ecological novelty have been extensively studied in plants but remained unclear in vertebrates owing to the rare polyploidy incidences. Here, a huge number of 3105 specimens in *Carassius* species complex including 2211 hexaploids and 894 tetraploids were sampled from 34 locations through mainland China. And hexaploids had wider geographic distribution than tetraploids especially in the areas with high altitude, high latitude and low annual precipitation. Then, an approximate 1050 bp *transferrin* (*tf*) fragments were amplified from all the samples, and 526 *tf* alleles were identified from a total of 37260 sequences at last. Intriguingly, higher nucleotide diversity of *tf* alleles in hexaploids than in tetraploids was revealed. Moreover, via phylogenetic analysis of *tf* alleles, potential origin center of *Carassius* species complex was deduced to be Yangtze River basin and hexaploids should undergo multiple independent polyploidy origins from sympatric tetraploids. These findings indicate that the hexaploids might possess stronger environmental adaptation and ecological novelty than the tetraploids, which provide an association paradigm of recurrent polyploidy and ecological context in polyploid vertebrates.

## Introduction

Polyploidy has been revealed to have significant potentials and evolutionary consequences for increasing allelic diversity, altering genomic complexity, introducing novel traits, and driving ecological transfiguration in plants^1^, because it has been found to be ubiquitous and to benefit from both physiological and genetic buffering to provide the raw materials for evolution and adaptation of diverse plants^3–5^. In comparison with the prevalent incidences in plants, polyploid species are rare in vertebrates^2^, but polyploidy events have been yet documented in a wide taxa including teleost fishes, amphibians and reptiles^3–6^, and polyploidy consequences are frequently associated with some unusual clonal reproduction modes, such as parthenogenesis, gynogenesis, hybridogenesis and kleptogenesis^7, 8^. Owing to the association with clonal reproduction without genetic recombination, polyploid vertebrates are generally considered to be evolutionary “dead-ends”^9^. Therefore, some key evolutionary enigmas, such as how polyploidy *per se* lead to ecological novelty and what extent the extant polyploids contribute to environmental adaptation, remain unknown in vertebrates.

Fishes are the most dominant and successful group of vertebrates in the world, and polyploidy events have been revealed to occur in numerous taxonomic orders including Cypriniformes, Salmoniformes, Perciformes, Siluriformes, Acipenseriformes, Gymnotiformes and Characiformes^4, 10^. In Cypriniformes, there are not only more than 250 recognized polyploid species across Asia, Europe, Africa and America, but also exist different ploidy of polyploids, such as triploids, tetraploids, hexaploids or even octoploids^4, 10, 11^. Especially in *Carassius* species complex of the genus *Carassius* that widely distribute across the Eurasian continent^12–14^, *Carassius auratus* with 100 chromosomes have been demonstrated as ancestral tetraploids that reproduce by bisexual reproduction^15, 16^, whereas *Carassius gibelio* with 156 or 162 chromosomes^17^ have been recognized as hexaploids that are able to reproduce by dual modes of unisexual gynogenesis and bisexual reproduction^3, 18^. Through evolutionary history analyses of two divergent *Dmrt1* genes, tetraploids were proposed to be formed via an early polyploidy event, and hexaploids were suggested to be resulted from ancestral tetraploids via recurrent polyploidy^11, 19^ Therefore, the rare extant case of ancestral tetraploids and recurrent hexaploids from the same evolutionary lineage makes the *Carassius* species complex as an ideal model to investigate adaptive evolution and ecological novelty of recurrent polyploidy in vertebrates.

In this study, we performed a field investigation to distinguish geographic distribution patterns of recurrent hexaploid *Carassius gibelio* and ancestral tetraploid *Carassius auratus* in *Carassius* species complex across a wide range throughout mainland China. Moreover, phylogenetic analysis and genetic diversity were performed by using *transferrin* (*tf*) alleles, because *tf* allele marker had been demonstrated to be particularly valuable for elucidating origin and evolutionary history of the polyploidy *Carassius* species complex^13, 20^ due to its polymorphisms especially in wild populations. Based on these data, we further explored the evolutionary implication for environmental adaptation and ecological novelty of repeated polyploidy from the ancestral tetraploid and recurrent hexaploid complex.

## Methods

### Sampling

**Figure 1:**
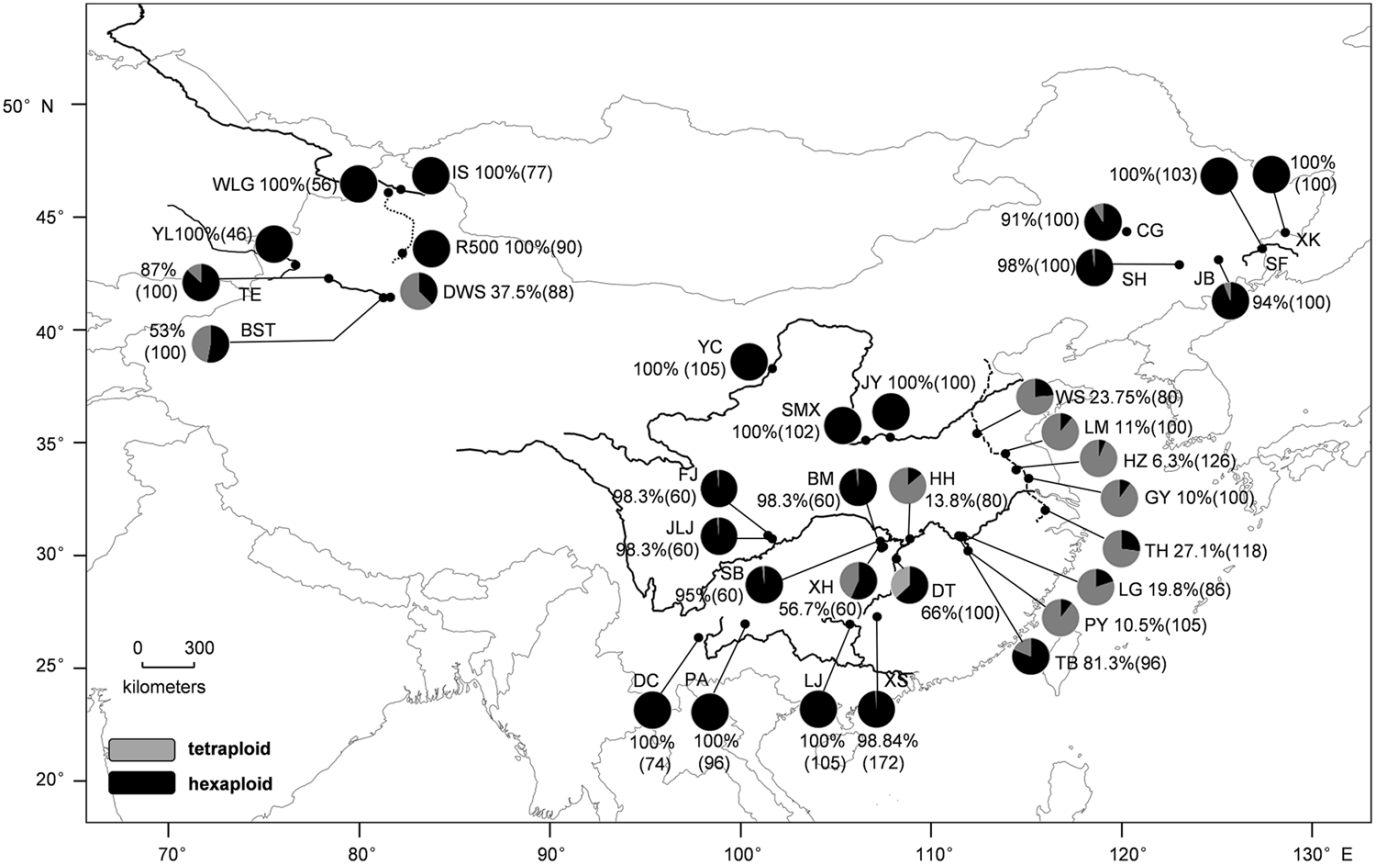
Geographical distribution of the 34 sampled populations. The detailed information of sampled sites is in Table S1. The proportions of hexaploids and tetraploids are indicated by the sizes of black and gray pie charts respectively. Percentage of hexaploids and sample size in bracket are given nearby. The map was created in ARCMAPv.9.3 (ESRI, Redlands, CA, USA) (http://www.esri.com/products).

A total of 3105 individuals of the *Carassius* species complex were collected widely from 34 sampled sites throughout mainland China. Details about all the samples and sampling sites are given in Table S1 and Fig. 1. For all specimens sampled, caudal fin and blood were obtained. Caudal fin was preserved in 100% ethanol for subsequent DNA extraction and PCR amplification. The deposited blood cells were then washed 2–3 times with 1 × phosphate buffered saline (PBS) solution and fixed in 75% ethanol for ploidy determination. All the specimens sampled were deposited in the Institute of Hydrobiology in China. All experiments in this research were performed according to the permit guidelines established by the Institute of Hydrobiology, Chinese Academy of Sciences, and the experimental protocols were approved by the animal care and use committee of Institute of Hydrobiology, Chinese Academy of Sciences.

### Ploidy determination and analysis of ploidy distribution pattern

We used high speed sorting flow cytometer FACSAriaTMIII (BD) to estimate ploidy levels for each sample by measuring the relative DNA content of their fixed blood cells following the documented instructions^21^. Chicken blood cells with known DNA content of 2.5 pg/nucleus were used as an internally quantitative standard for each individual flow cytometry profile. The sampled blood cells of each individual were mixed with chicken blood cells and fixed in 70% pre-cooled ethanol overnight at 4 °C. The mixed cells were washed 2–3 times in 1 × PBS and then resuspended in the solution including 0.5% pepsin and 0.1 M HCl. DNA was stained with propidium iodide solution (40 g/mL) for 1–3 h at room temperature in the dark. Each sample with at least 10000 cells was measured in triplicate.

### Analysis for ploidy distribution pattern

In our study, only hexaploids and tetraploids were observed in all the populations and their proportions were calculated subsequently. To evaluate the distribution pattern of the two ploidy forms in *Carassius* species complex, all sampling locations were divided into eight geographic areas according to the drainage systems through mainland China, including upper basin of Pearl River (LJ), upper basin of Yangtze River (DC, PA, FJ, JLJ), middle basin of Yangtze River (XS, DT, BM, XH, SB, TB, HH), lower basin of Yangtze River (LG, TH, PY), Yellow River (YC, JY, SMX), Jing-Hang Grand Canal (WS, HZ, LM, GY), northeast of China (XK, JB, SF, CG, SH), and northwest of China (BST, DWS, TE, R500, IS, WLG, YL). To examine the effect of geographic/climatic factors on distribution pattern of *Carassius* species complex, all the populations were assigned into four groups based on the proportions of hexaploids, including low hexaploid frequency group (hexaploid percentage ranging from 0 to 50%), sub-high hexaploid frequency group (hexaploid percentage ranging from 50% to 85%), high hexaploid frequency group (hexaploid percentage ranging from 85% to 99%) and all-hexaploid group (hexaploid percentage is 100%). The geographic/climatic factors used for analysis are longitude, latitude, altitude, average annual temperature (abbreviated as T_mean_) and annual precipitation. The data of temperature, altitude and precipitation were obtained from WorldClim database^22^ and the data of sampling points were extracted using ARCMAPv.9.3 (ESRI, Redlands, CA, USA).

Constrained and partial canonical ordinations were used to test the influence of the above geographic/climatic variables on hexaploid and tetraploid distribution. Detrended correspondence analysis was performed to determine the appropriate type of model for direct gradient analysis^23^. Detrended correspondence analysis indicated that a linear model (gradient lengths < 2 standard units) would best fit the data of our study and redundancy analysis was performed subsequently.

The percentages of hexaploids and tetraploids were log (*x* + 1) transformed prior to analysis. Geographic/climatic variables that did not confirm the normality assumption were transformed using natural logarithms, while the other variables that were normally distributed were not transformed^24^. Variables with variation inflation factors > 20 were removed from the analysis to avoid high colinearity^25^. A Monte Carlo test with 9,999 permutations was used to determine the significance under the null hypothesis, which shows no relationship between species and environmental variables. The environmental factors showing rejection to null hypothesis (p < 0.05) in Monte Carlo test could be selected in redundancy analysis^26^ using the software CANOCO for Windows 4.5 version^27^. Based on the redundancy analysis data, the hexaploid group regression function, 0.833 × altitude + 0.681 × latitude − 0.322 × precipitation, was obtained. Then the hexaploid group index of 237 localities containing the 34 sampling points in this study were calculated and interpolated using the ordinary kriging method^28^ implemented in ARCMAPv.9.3 (ESRI, Redlands, CA, USA) to predict the distribution pattern of two ploidy forms throughout mainland China.

### Nucleotide sequencing and sequence analysis

Genomic DNA was extracted from fin clips by DNeasy Blood & Tissue Kit (QIAGEN) following the manufacturer’s protocol. We used the following primers *tf*-F (CTCCTCAAAGAGCCTCGCCAT) and *tf*-R (TACACCTGGCCACCATCAACTG)^13^ to amplify an approximate 1050 bp fragment between the 7th exon to 10th exon of *tf* gene^13, 29^. PCR products were cloned into pMD-19T vector (Takara), and 12 positive clones were sequenced for each sample. M13F (−47) and M13R (−48) primers were used for bidirectional Sanger sequencing on 3730XL-96 platform (ABI).

Sequence alignments and information about nucleotide variation of *tf* alleles were identified using MEGA 7.0^30^. Alleles of *tf* were generated by DnaSP 5.10^31^ software and then arranged according to their frequency. Nucleotide diversity (π) of both the 22 populations with two ploidy forms and eight geographic areas were evaluated using Arlequin version 3.5^32^. Phylogenetic analysis of *tf* alleles was conducted using Bayesian inference (BI) in MRBAYES 3.1.2^33^ and maximum-likelihood (ML) in RA_X_ML^34^. We selected the best fit nucleotide substitution model using MODELTEST version 3.7^35^. For BI tree, four independent Markov chain Monte Carlo (MCMC) chains were simultaneously run for 20,000,000 generations with sample frequency of 1000 generations. The first 25% of the trees were discarded as burn-in and the remaining tree samples were used to generate a consensus tree. For ML tree, nodal support value was assessed from 100 nonparametric bootstrap replicates. In addition, to investigate the relationship between *tf* alleles, a median-joining network was built using Network4.6.0.0^36^.

To estimate divergence times in *Carassius* species complex, an uncorrelated relaxed molecular clock approach was implemented in Beast 1.7.5^37^. GTR+I+G was selected as the best fit model of evolution using MODELTEST software of version 3.7^35^. The divergence time (11.11–9.14 million years ago (Mya))^38^ between *Cyprinus carpio* and *Carassius* species complex was used as the calibration. The Markov Chain Monte Carlo (MCMC) analyses were run for 100,000,000 generations with sample frequency of 1,000 generations. Tracer v1.5 (http://beast.bio.ed.ac.uk/Tracer) was used to ensure adequate mixing of the MCMC with effective sample sizes (ESS) above 100. The plausible trees were summarized in the maximum clade credibility (MCC) tree after discarding first 60% of sampled generations by Tree Annotator v1.7.5, and then the results were visualized and edited in FigTree 1.4 (http://beast.bio.ed.ac.uk/FigTree).

## Results

### Wider geographic distribution pattern in hexaploids than in tetraploids

A total of 3105 individuals of *Carassius* species complex were sampled from 34 different geographic locations through mainland China. Among the sampled specimens, 2211 (71.2%) were examined as hexaploids and 894 (28.8%) as tetraploids by flow cytometry^13, 21^ (Table S1). Among the 34 sampled locations, there were 12 all-hexaploid populations and 22 sympatric populations of both hexaploids and tetraploids, whereas no all-tetraploid populations were detected (Table S1 and Fig. 1). Intriguingly, all-hexaploid and highly biased-hexaploid populations were majorly distributed in northeast of China, northwest of China, Yellow River, upper basins of Pearl River and Yangtze River, whereas both hexaploids and tetraploids were mainly observed to overlap in most populations of Jing-Hang Grand Canal and middle/lower basins of Yangtze River (Table S1 and Fig. 1). The comprehensive survey data indicate a significant differential distribution pattern of *Carassius* species complex that the hexaploids have wider geographic distribution than the tetraploids.

### Association between geographic/climatic variables and the differential distribution pattern in *Carassius* species complex

**Figure 2:**
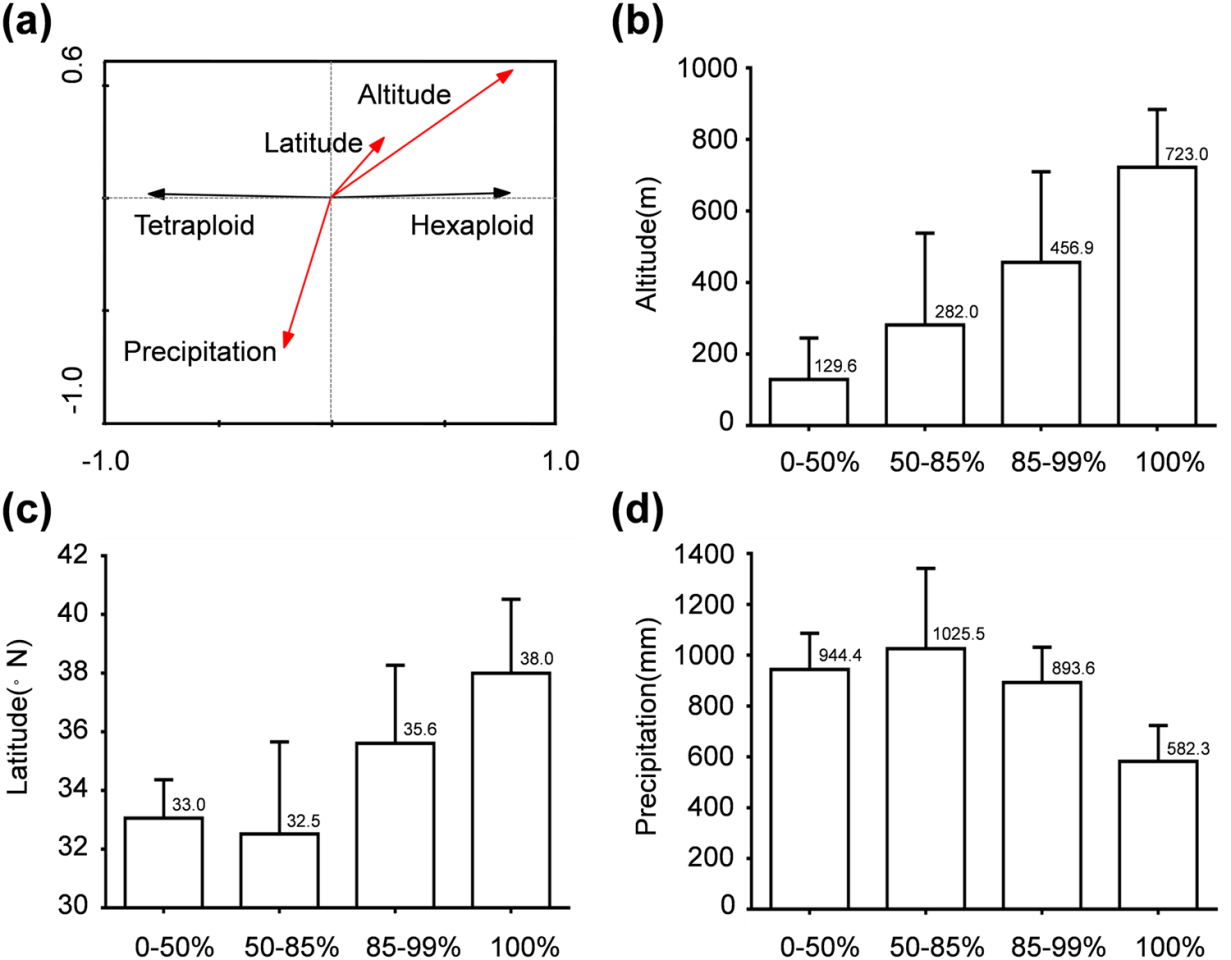
(**a**) The redundancy analysis on association between geographic/climatic variables and differential distribution pattern of hexaploids and tetraploids. Hexaploid and tetraploid are shown by black arrows, while altitude, latitude, and precipitation are shown by red arrows. Diagrams between different hexaploid frequencies and the correlated geographic/climatic variables including altitude (**b**), latitude (**c**) and precipitation (**d**). The X-axes indicate the hexaploid frequencies, and Y-axes indicate values of the correlated geographic/climatic variables.

A total of 5 geographic/climatic variables including altitude, longitude, latitude, average annual temperature (T_mean_) and annual precipitation were used to exam the association between geographic/climatic variables and the differential distribution pattern (Table S2). Via redundancy analysis, differential distribution pattern in *Carassius* species complex was revealed to be significantly associated with altitude (inflation factor = 2.35, p = 0.0001), latitude (inflation factor = 12.07, p = 0.024) and annual precipitation (inflation factor = 14.73, p = 0.049), whereas it was unrelated to the other variables including T_mean_ and longitude (Table 1). And, the hexaploid frequency was positively correlated to altitude and latitude and negatively related to annual precipitation, while the tetraploid frequency was negatively related to altitude and latitude and positively correlated to annual precipitation (Fig. 2a). Moreover, the examined 34 populations were respectively assigned to four different hexaploid frequency groups (0–50%, 50–85%, 85–99%, 100%) to clarify relative association between the three significant geographic/climatic variables and the distribution pattern (Table S2). Significantly, only 10.2% of tetraploids but 89.8% of hexaploids exist in the populations at altitude higher than 456.9 m, and all-hexaploid populations generally distribute at the over 723.0 m elevation (Fig. 2b). Moreover, 89.3% of hexaploids spread in the populations with latitude over 35.6°N, while only 10.7% of tetraploids exist in this latitude range, and all-hexaploid groups tend to occur in drainages of high latitude with 38.0°N (Fig. 2c). Additionally, a total of 77.9% of hexaploids tend to inhabit in drainage basins with annual precipitation less than 893.6 mm, while only 22.1% of tetraploids exist in the populations with the same range of annual precipitation, and all-hexaploid groups tend to occur in the area with low annual precipitation under 582.3 mm (Fig. 2d). These data indicate that hexaploids might be more tolerant to harsh geographic/climatic variables than tetraploids.

**Table 1:** Redundancy analysis results of 5 geographic/climatic variables that are associated with differential geographic distribution of hexaploids and tetraploids.

Variable	Inflation factor	P-value
altitude	2.35	0.0001*
longitude	4.37	0.108
latitude	12.07	0.024*
T_mean_	3.47	0.068
Annual precipitation	14.73	0.049*

**Figure 3:**
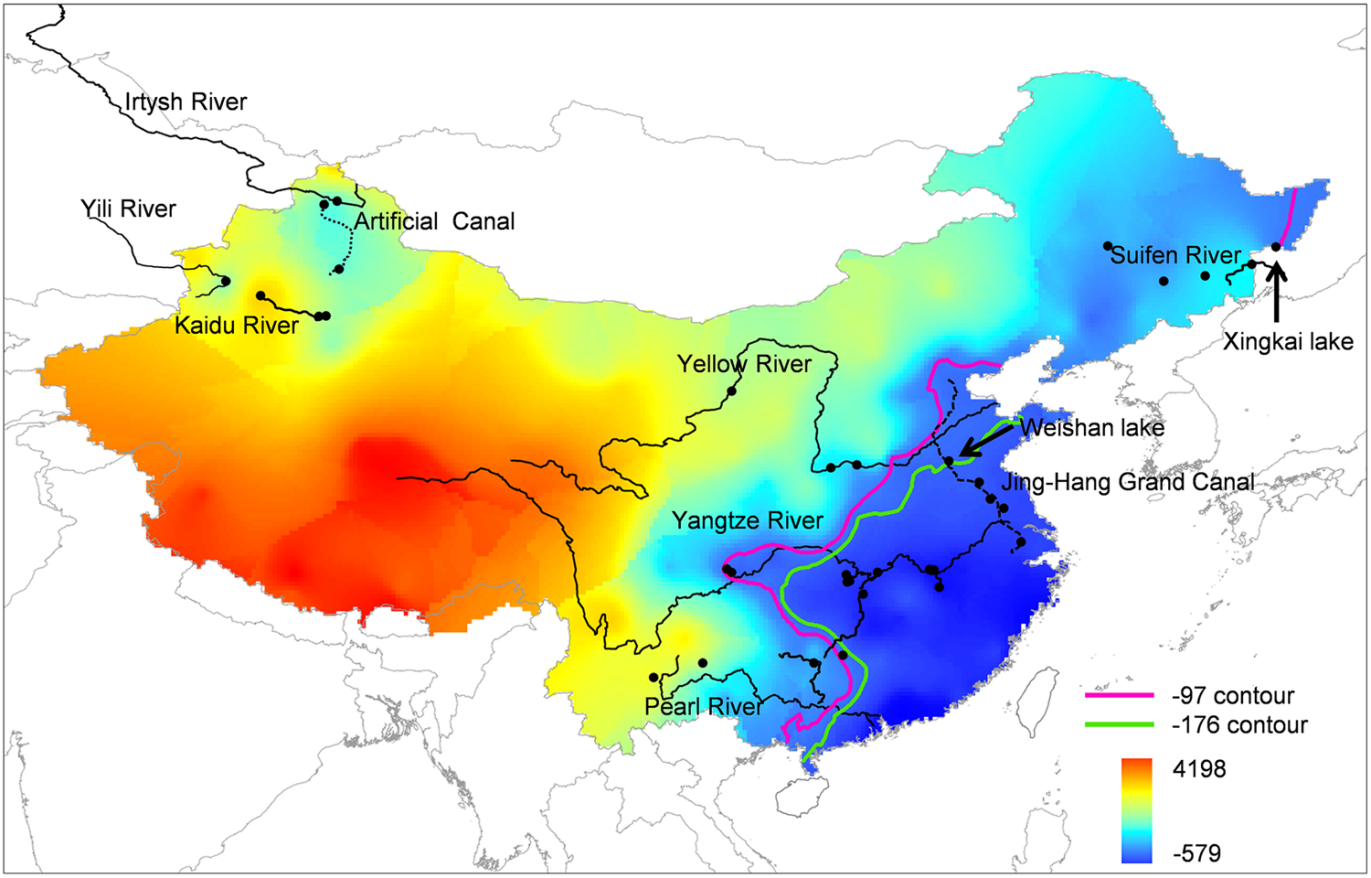
A predicted map of hexaploid frequency distribution throughout mainland China by using hexaploid group indexes. As hexaploid group indexes were of significantly difference (p = 0, < 0.05) among three assigned hexaploid frequency groups, the lowest value of all-hexaploid frequency group at Xingkai lake (−97) and the highest value of high frequency (51–99%) hexaploid group at Weishan lake (−176) were respectively used as critical values of all-hexaploid group and high frequency (51–99%) hexaploid group through discriminant analysis. The purple −97 contour line stands for the predicted boundary between all-hexaploids and high frequency (51–99%) hexaploids. The green −176 contour line represents the predicted boundary between high frequency (51–99%) hexaploids and low frequency (0–50%) hexaploids. −579 and 4198 indicate the lowest and highest values of predicted hexaploid group indexes respectively. The map was created in ARCMAPv.9.3 (ESRI, Redlands, CA, USA) (http://www.esri.com/products).

Based on the redundancy analysis data in Fig. 2a, a hexaploid frequency group regression function, 0.833 × altitude + 0.681 × latitude − 0.322 × precipitation was obtained. To reveal further association between the ploidy distribution and the habitats, different parameters of three geographic/climatic variables in 237 localities throughout China were used to calculate the corresponding hexaploid group indexes (Table S3). We used hexaploid group indexes at Xingkai lake (−97) and Weishan lake (−176) as critical values of all-hexaploid group and low frequency (0–50%) hexaploid group through discriminant analysis, because they were closest to the lowest value of all-hexaploid frequency group and the highest value of low frequency hexaploid group respectively (Table S3). Thereby, a predicted map of hexaploid frequency distribution throughout mainland China was constructed (Fig. 3). When the hexaploid group index value of a population is higher than −97, this population would tend to be almost all-hexaploids. And, when the hexaploid group index value of a population is lower than −176, the population would tend to contain low frequency hexaploids (less than 50%). While the index of a population is between −97 and −176, this population would tend to have higher frequency of hexaploids (more than 50%). In the predicted map, all-hexaploid and highly biased-hexaploid populations were majorly distributed in northeast, northwest, upper basins of Yangtze River, Pearl River and Yellow River, whereas low frequency hexaploids were mainly observed in Jing-Hang Grand Canal and middle/lower basins of the Yangtze River (Fig. 3). Thus, actual distribution pattern of *Carassius* species complex (Fig. 1) is nearly concordant with the predicted map (Fig. 3), which indicates that geographic distribution of hexaploids is wider than that of tetraploids through mainland China. And hexaploids distribute predominantly in the areas of high altitude, high latitude or low precipitation (Figs 1, 2 and 3).

However, a few of exceptional populations with tetraploids were also observed from several lakes, such as Bositeng lake, Dawusong lake and Tian’e lake in northwest, as well as Chagan Lake, Jingbo Lake and Songhua Lake in northeast (Fig. 1). These exceptions might be resulted from human activities, as *Carassius* species complex was previously introduced from middle and lower basins of the Yangtze River to these lakes, especially Bositeng lake and Dawusong lake^39^.

### Higher nucleotide diversity in hexaploids than in tetraploids

Firstly, *tf* fragments ranging from 1002 bp to 1108 bp were amplified to clarify their nucleotide differences among the 3105 samples. A total of 37260 clones were sequenced and 526 *tf* alleles were distinguished at last. Among the 526 alleles, 99 alleles (*tf*1–*tf*99) with occurrence frequencies ranging from 27.99% to 0.04% were shared by hexaploids and tetraploids, and 331 alleles (*tf*100–*tf*430) with occurrence frequencies ranging from 0.46% to 0.02% were detected only in hexaploids (Fig. S1), whereas only 96 alleles (*tf*431–*tf*526) with occurrence frequencies ranging from 0.38% to 0.02% were identified only in tetraploids (Fig. S1).

**Figure 4:**
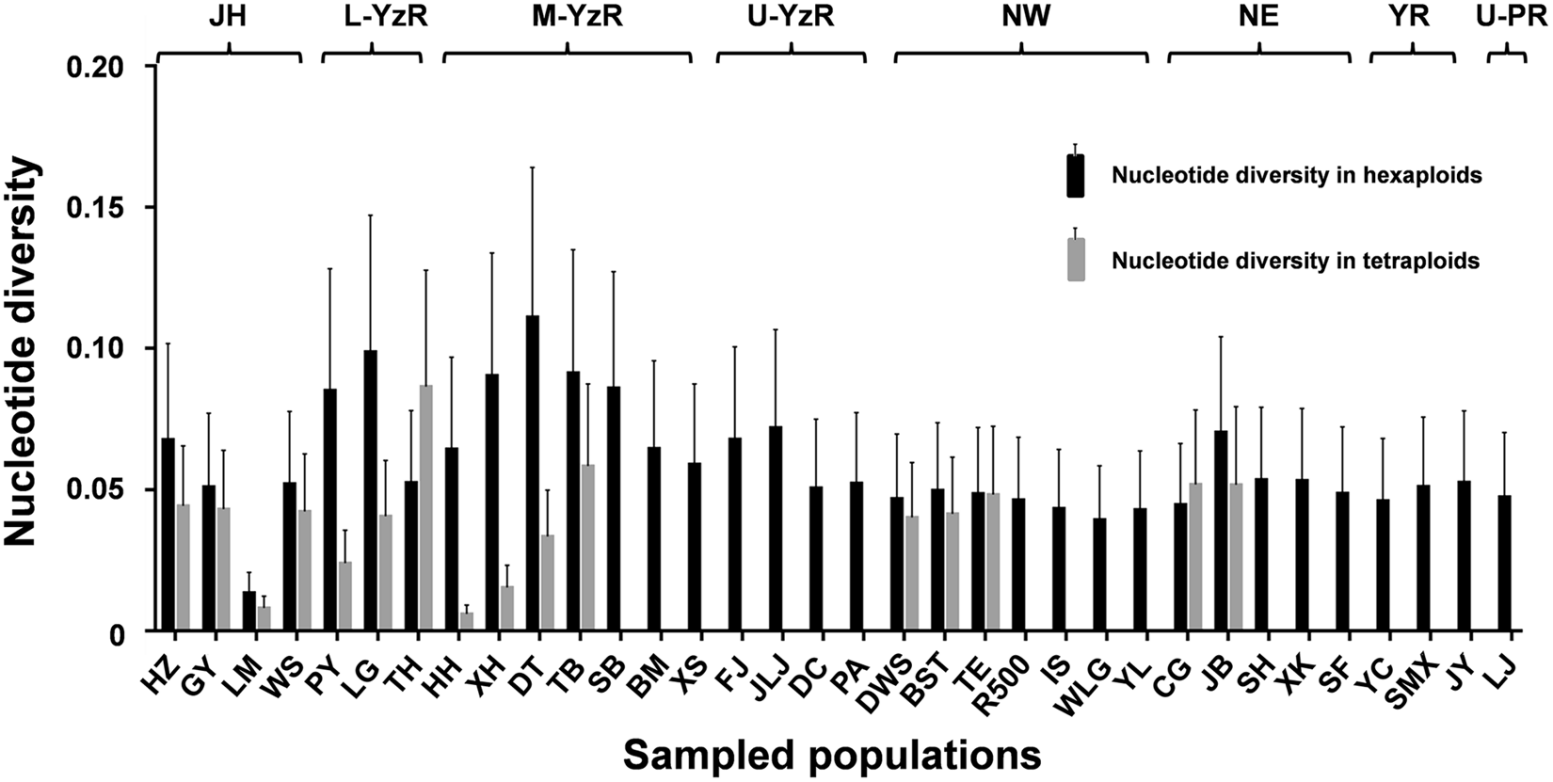
Nucleotide diversity of *tf* alleles in hexaploids (black) and tetraploids (grey). The X-axis shows the abbreviation of each sampled location, and Y- axis indicates value of nucleotide diversity. Geographic areas of the sampled locations are indicated by curly braces on the top. JH, Jing-Hang Grand Canal; L-YzR, lower basin of Yangtze River; M-YzR, middle basin of Yangtze River; U-YzR, upper basin of Yangtze River; NW, northwest of China; NE, northeast of China; YR, Yellow river; U-PR, upper basin of Pearl River.

Furthermore, we analyzed and compared nucleotide diversities of these *tf* alleles between hexaploids and tetraploids among the 34 sampled populations (Table S4). The total nucleotide diversity of hexaploids (0.0629 ± 0.0300) is higher than that of tetraploids (0.0546 ± 0.0261) in all populations. Moreover, in the 16 populations with enough hexaploids and tetraploids (at least five specimens of each ploid form), the mean value of nucleotide diversities in hexaploids are generally higher than those of sympatric tetraploids except the populations of Taihu lake and Chagan lake (Fig. 4). The higher nucleotide diversity in hexaploids than in tetraploids implicates that hexaploids might have more advantages than tetraploids, as higher nucleotide diversity is commonly able to provide more raw materials for new gene arising and adaptive evolution.

### Phylogenetic relationship and evolutionary history of *tf* alleles

**Figure 5:**
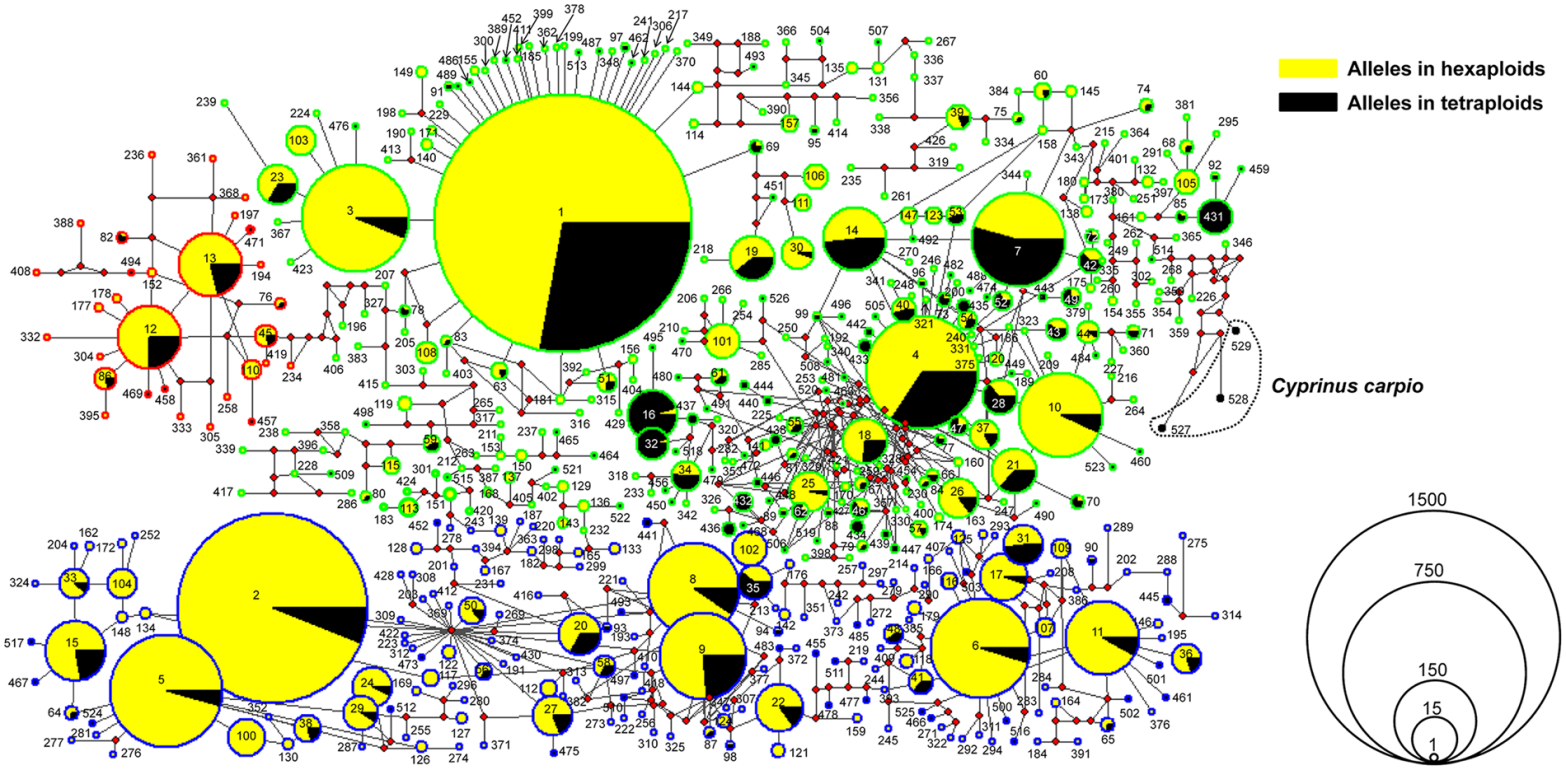
Median-joining network of 526 *tf* alleles identified from *Carassius* species complex. Three *tf* alleles from *Cyprinus carpio* are used as outgroup. Circles represent different alleles and their corresponding occurrence frequency in all sampled populations. Lineage A, B and C are exhibited in red, green and blue of border colors respectively, which also stand for phylogroups A, B and C in Fig. S2. Yellow and black colors inside the circle show the percentage of hexaploid and tetraploid respectively. Allele codes are denoted inside or beside the circles. Solid red spots represent un-sampled or predicted alleles.

Three major lineages were revealed via median-joining network analysis based on the identified 526 *tf* alleles (Fig. 5). Lineage A included 30 *tf* alleles, in which 6 *tf* alleles were shared by hexaploids and tetraploids, and 19 and 5 *tf* alleles were respectively detected only in hexaploids and in tetraploids (Fig. 5). Meanwhile, *tf* alleles of lineage A mainly existed in the samples of Yangtze River basin, Jingbo lake and Yili River. A total of 317 *tf* alleles were grouped into lineage B, in which 63 *tf* alleles were shared by two ploidy forms, and 190 and 64 *tf* alleles were respectively detected only in hexaploids and in tetraploids (Fig. 5). The highest occurrence frequency of *tf*1 (27.6%) was not only shared by hexaploids and tetraploids but also detected from all populations throughout mainland China (Fig. 5). The lineage C included 179 *tf* alleles, in which 30 *tf* alleles were detected in hexaploids and tetraploids, 122 *tf* alleles only in hexaploids, and 27 *tf* alleles only in tetraploids. The high frequency occurrence of alleles (*tf*2 and *tf*5) were also found in all sampled populations. The high frequency occurrence and extensive distribution implies that *tf*1, *tf*2 and *tf*5 might be ancient *tf* alleles and reflect a common origin of *Carassius* species complex.

**Figure 6:**
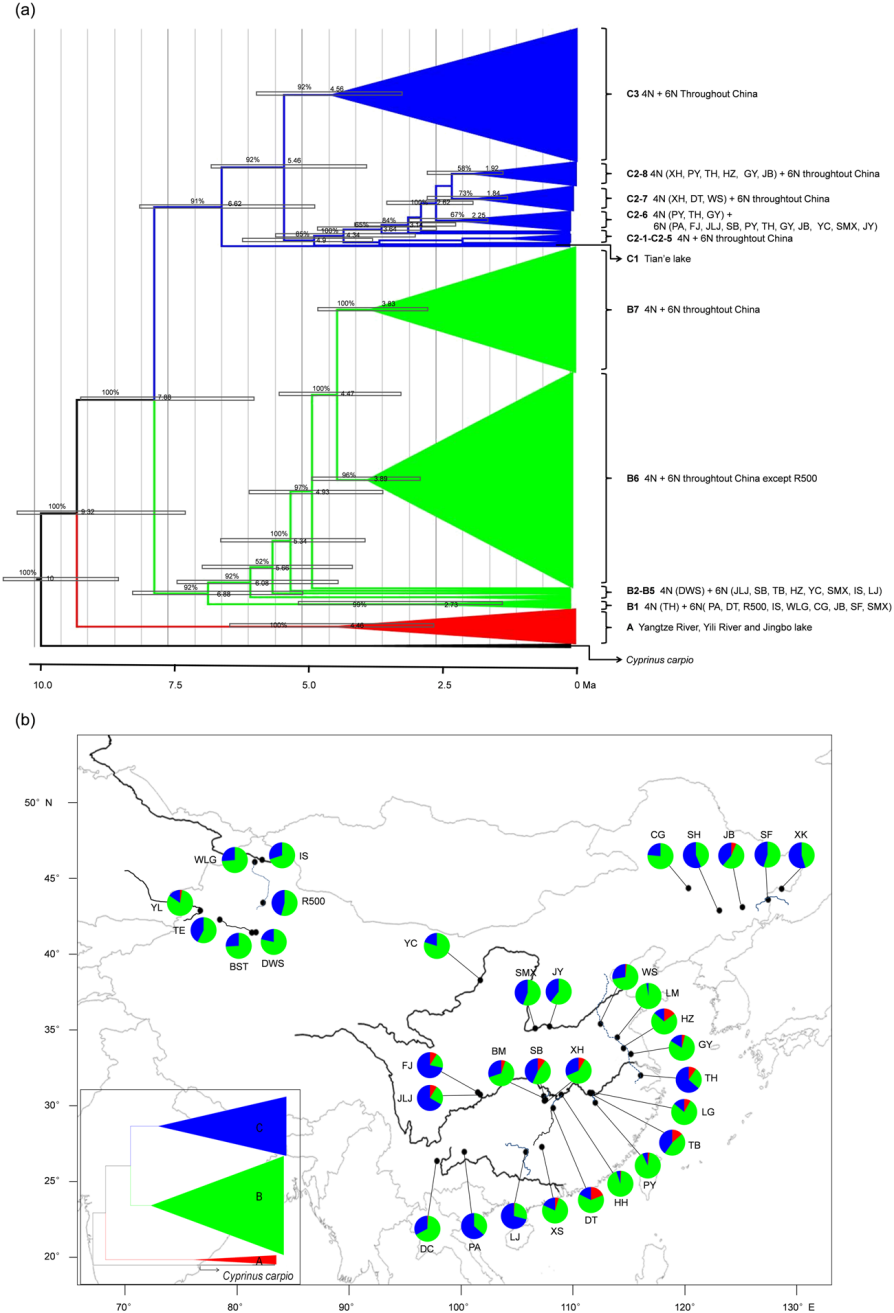
Simplified time-calibrated phylogeny tree (**a**) and relative frequency of three different lineages in 34 sampled populations (**b**). Values above branches indicate Bayesian posterior probabilities, and divergence times are shown near each node. *Tf* allele of *Cyprinus carpio* is used as outgroup. *Tf* alleles in each lineage are described in detail in Fig. S2. 4N and 6N are the abbreviation of tetraploids and hexaploids respectively. Each lineage is uniquely colored and is matched with the color of pie-diagrams in Fig. 5. The map was created in ARCMAPv.9.3 (ESRI, Redlands, CA, USA) (http://www.esri.com/products).

Based on the 526 *tf* alleles, a Bayesian tree was generated (Fig. S2), and similar topology structure with MJ network was revealed. Once again, these *tf* alleles of hexaploids and tetraploids were intermixed in lineage A, lineage B and lineage C, and numerous alleles were shared by both hexaploids and tetraploids within each lineage (Fig. S2). Furthermore, a simplified time-calibrated phylogenetic tree of these *tf* alleles was constructed. As shown in Fig. 6a, lineage A was the earliest split at about 9.32 Mya, and lineage B and C were diverged around 7.88 Mya. In lineage B, sublineage B1 from 10 sampled populations and sublineage B2-B7 from individuals throughout China were separated around 6.88 Mya. In lineage C, sublineage C1 with only one *tf* allele (*tf*418) from Tian’e lake was divided around 6.62 Mya, which might be the result of human activity. Sublineage C2 and sublineage C3 occurred in all populations were split around 5.46 Mya. Significantly, the repeated polyploidy traces during the expansion of *Carassius* species complex could be also detected from some branch topology structures with high support in lineage C. For example, sublineage C2-6, C2-7 and C2-8 with both hexaploids and tetraploids were split at about 2.25 Mya, 1.84 Mya and 1.92 Mya respectively (Fig. 6a). The above data suggest that hexaploids might originate from tetraploids independently via multiple autopolyploidy events in *Carassius* species complex, because hexaploids and tetraploids are intermixed in the same lineages and they share the same alleles (Fig. 6a and Fig. S2). In addition, relative frequencies of three different lineages in all sampled populations were presented (Fig. 6b). The first separated older lineage A mainly appearing in most populations of Yangtze River basin (Fig. 6) and higher nucleotide diversities in middle/lower basins of Yangtze River than in other areas (Fig. S3) suggested that Yangtze River basin might be the potential origin center of *Carassius* species complex, and then the *Carassius* species complex distribute to other rivers and lakes throughout China.

## Discussion

In comparison with ubiquitous existence in plants^40–43^, polyploidy incidences in animals are rare, especially in vertebrates including fishes, amphibians and reptiles^3, 4, 6, 19, 44–46^. However, our current study in *Carassius* species complex may provide some novel knowledge about adaptive evolution and ecological novelty of recurrent polyploidy in vertebrates from the natural evolutionary coexistence system of ancestral tetraploids and recurrent hexaploids. Through this study, we had distinguished 2211 hexaploids and 894 tetraploids in 3105 specimens of *Carassius* species complex sampled from 34 locations in mainland China (Fig. 1 and Table S1), and observed a close association between differential distribution pattern of hexaploids and tetraploids and geographic/climatic variables including altitude, latitude and annual precipitation (Fig. 2 and Table 1), in which hexaploids had wider distribution than tetraploids especially in the areas with higher altitude, higher latitude and lower annual precipitation (Fig. 3). Moreover, we unearthed and analyzed various *tf* alleles from the sampled hexaploids and tetraploids, and commonly revealed higher nucleotide diversity in hexaploids than in tetraploids. These data indicate that the hexaploids might possess stronger environmental adaptation and ecological novelty than the ancestral tetraploids. Intriguingly, similar geographic distributions and high genetic diversity have been also observed in some regional investigation of *Carassius* species complex in East Asia^11, 13^. Similar to the polyploidy in plants, which has been revealed to have significant evolutionary consequences for increasing genetic diversity, genomic complexity and ecological adaption^1, 47–50^, hexaploids indeed possess wider geographic distribution and higher genetic diversity than the ancestral tetraploids.

In addition, this study further helps us understand the evolutionary history and trajectory of different ploidy forms in *Carassius* species complex. In *Carassius* species complex, the ancestral tetraploids with 100 chromosomes and the recurrent hexaploids with about 150 chromosomes^15, 16^ were extensively found in the Eurasian continent^12–14, 51, 52^, and a few octoploids with about 200 chromosomes were occasionally recorded in some natural habitats and artificial populations^39, 53^. In this survey, only hexaploids and tetraploids were identified, and their phylogenetic relationship and evolutionary history (Figs S2 and 6) were analyzed from the identified 526 diverse *tf* alleles. As revealed by Figs 5 and 6, three major lineages were presented, and numerous alleles were shared by both hexaploids and tetraploids within each lineage. Based on the previous hypothesis that polyploidy occurrence might be related to glaciations and quaternary climatic changes^38, 54^, our current study further suggests that Yangtze River basin should be the refuge during glaciations and potential origin center of *Carassius* species complex, and then the *Carassius* species complex radiates across East Asia during this expansion, as the older lineage A mainly consists of populations from Yangtze River basin (Fig. 6) and the highest nucleotide diversity also is from the populations of Yangtze River basin (Fig. S3). The fossil of *Carassius* in Pliocene epoch (5.3–2.6 Mya) in north of China (Yushe, Shanxi province)^55^ might provide evidence for expansion of *Carassius* species complex. Moreover, hexaploids might originate from tetraploids independently via multiple autopolyploidy events in *Carassius* species complex, because hexaploids and tetraploids are intermixed in the same lineages and they share the same alleles (Fig. 6 and Fig. S2).

The evolutionary success of recurrent polyploidy has been mainly attributed to the diploidization process followed by polyploidy^56–58^. Based on the current findings and previous reports^3, 59, 60^, we believe that extant hexaploids in *Carassius* species complex are entering an evolutionary trajectory of diploidization or have undergone diploidization, because normal meiosis completion, multiple modes of unisexual gynogenesis and sexual reproduction, and extra microchromosomes for male determination, have been observed in some gynogenetic clones of hexaploid *Carassius gibelio* ^59–61^. However, recurrent polyploidy is not ceased in *Carassius* species complex as occasional octoploids have been also detected in some natural drainage systems and artificially proliferated populations^31, 39^. Thus the *Carassius* species complex provides an ideal system to better understand the consequences of recurrent polyploidy and fills in the huge gaps across the spectrum of ecology, genetics and evolution biology in polyploid vertebrates.

## Electronic supplementary material

Supplementary information
