# Supplementary material for: Wider geographic distribution and higher diversity of hexaploids than tetraploids in Carassius species complex reveal recurrent polyploidy effects on adaptive evolution
Source: Sci Rep. 2017 Jul 14;7:5395. doi: 10.1038/s41598-017-05731-0 (PMC5511294; doi:10.1038/s41598-017-05731-0)
Supplement: Supplementary file 1 — Supplementary information [file 41598_2017_5731_MOESM1_ESM.pdf]

Supplementary information

Wider geographic distribution and higher diversity of hexaploids than tetraploids in *Carassius* species complex reveal recurrent polyploidy effects on adaptive evolution

Xiao-Li Liu, Fang-Fang Jiang, Zhong-Wei Wang, Xi-Yin Li, Zhi Li, Xiao-Juan Zhang, Fan Chen, Jian-Feng Mao, Li Zhou, Jian-Fang Gui

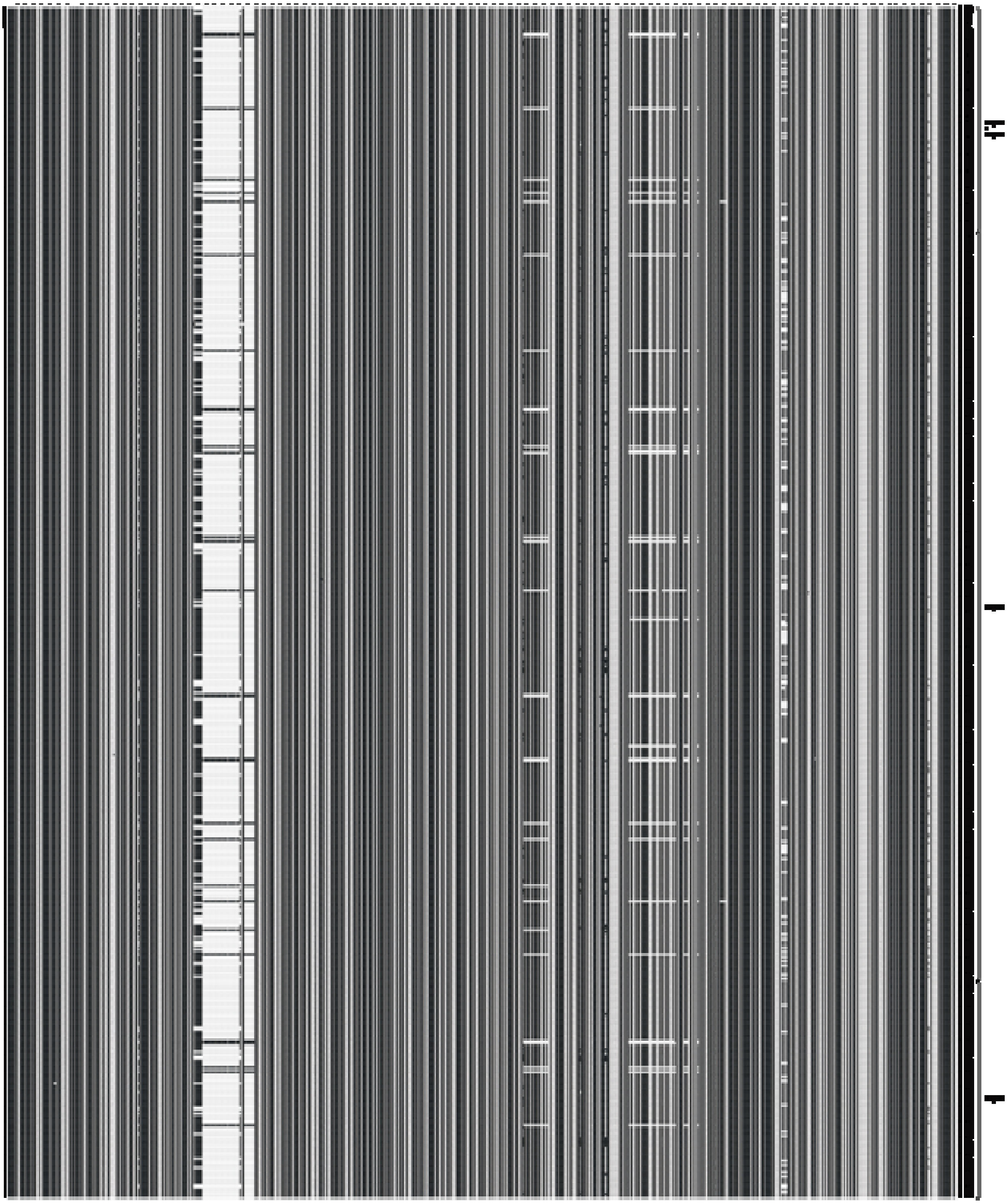

Supplementary Figure 1. Nucleotide sequences of the identified 526 *tf* alleles. The same bases with threshold value of 80% were showed with black areas. Gaps (-) are introduced to optimize identity. The occurrence frequencies are shown at the end of each sequence.

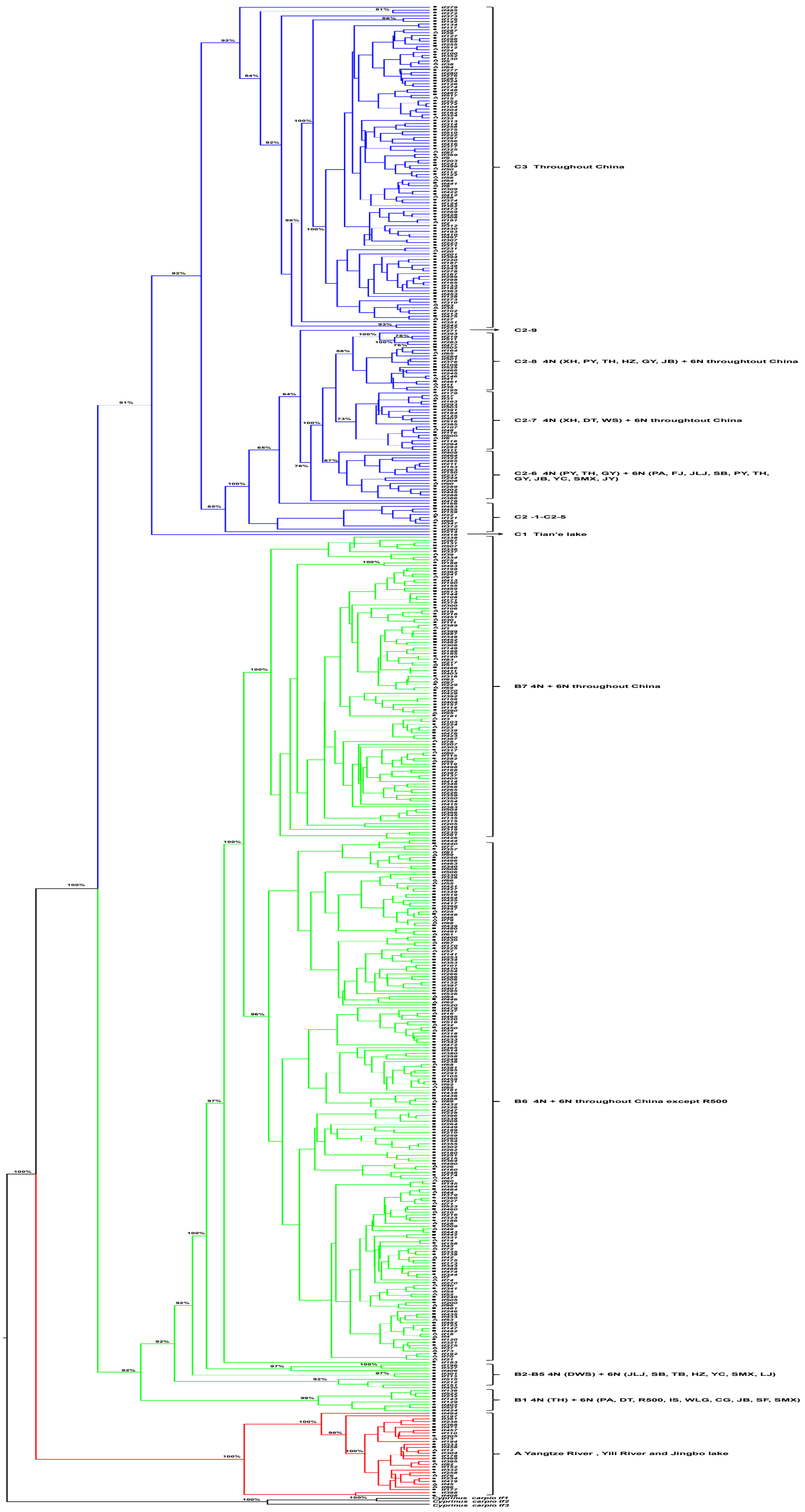

Supplementary Figure 2. Bayesian tree of 526 *tf* alleles in *Carassius* species complex. Bayesian posterior probabilities (BPP) of >50% are shown around nodes. Major lineages and sublineages are distinguished by different colors. Lineage A, B and C are exhibited in red, green and blue respectively. Solid circles and squares indicate alleles only identified from hexaploids and tetraploids respectively. Hollow triangles refer to shared alleles between the two ploidy forms. *Tf* alleles in *Cyprinus carpio* are used as outgroup.

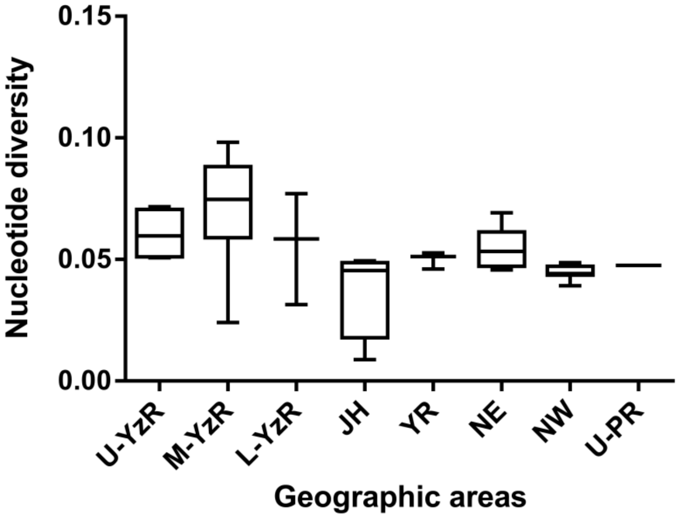

Supplementary Figure 3. Nucleotide diversity of *tf* alleles in *Carassius* species complex of 8 different geographic areas. The X-axis shows geographic areas of the sampled locations, and Y- axis indicates value of nucleotide diversity. U-YzR, upper basin of Yangtze River; M-YzR, middle basin of Yangtze River; L-YzR, lower basin of Yangtze River; JH, Jing-Hang Grand Canal; YR, Yellow river; NE, northeast of China; NW, northwest of China; U-PR, upper basin of Pearl River.

Supplementary Table 1. The sampled locality and its abbreviation (Abb), the divided geographic area, the sampled number (N) and its hexaploid percentage (%).

Code	Sampled locality and the Abb	Geographic area	Sampled number	Hexaploid percentage
1	Hongze Lake, Sihong county, Jiangsu (HZ)	Jing-Hang Grand Canal	126	6.30%
2	Gaoyou Lake, Gaoyou county, Jiangsu (GY)		100	10%
3	Luoma Lake, Suyu district of Suqian, Jiangsu (LM)		100	11%
4	Weishan Lake, Weishan county, Shandong (WS)		80	23.75%
5	Poyang Lake, Duchang county, Jiangxi (PY)	Lower Yangtze River	105	10.50%
6	Longgan Lake, Huangmei county, Hubei (LG)		86	19.80%
7	Taihu Lake, Wuxi city, Jiangsu (TH)		118	27.10%
8	Honghu Lake, Honghu county, Hubei (HH)	Middle Yangtze River	80	13.80%
9	Xihu Lake, Jinshi county, Hunan (XH)		60	56.70%
10	Dongting Lake, Xiangyin county, Hunan (DT)		100	66%
11	Taibai Lake, Huangmei county, Hubei (TB)		96	81.30%
12	Shanbo Lake, Anxiang county, Hunan (SB)		60	95%
13	Beimin Lake, Jinshi county, Hunan (BM)		60	98.30%
14	Xiaoshui, Shuangpai county, Hunan (XS)		172	98.84%
15	Fujiang, Hechuan district, Chongqing (FJ)	Upper Yangtze River	60	98.30%
16	Jialingjiang, Beibei district, Chongqing (JLJ)		60	98.30%
17	Dianchi, Chenggong district of Kunming, Yunnan (DC)		74	100%
18	Puan, Puan county, Guizhou (PA)		96	100%
19	Dawusong Lake, Heshuo county, Xinjiang (DWS)	Northwest	88	37.50%
20	Bositeng Lake, Heshuo county, Xinjiang (BST)		100	53%
21	Tian’e Lake, Hejing county, Xinjiang (TE)		100	87%
22	500 reservoir, Fukang city, Xinjiang (R500)		90	100%
23	IrtySh River, Aletai district, Xinjiang (IS)		77	100%
24	Wulungu Lake, Aletai district, Xinjiang (WLG)		56	100%
25	Yili River, Gongliu county, Xinjiang (YL)		46	100%
26	Chagan Lake, Qianguo county, Jilin (CG)	Northeast	100	91%
27	Jingbo Lake, Ningan county, Heilongjiang (JB)		100	94%
28	Songhua Lake, Jiaohe city, Jilin (SH)		100	98%
29	Xingkai Lake, Mishan city, Heilongjiang (XK)		100	100%
30	Suifen River, Suifenhe city, Heilongjiang (SF)		103	100%
31	Yellow river, Xingqing district of Yinchuan, Ningxia (YC)	Yellow River	105	100%
32	Yellow river, Hubin district of Sanmenxia, Henan (SMX)		102	100%
33	Yellow river, Jiyuan city, Henan (JY)		100	100%
34	Lijiang, Xing'an county, Guangxi (LJ)	Upper Pearl River	105	100%

Supplementary Table 2. The sampled populations, hexaploid percentages, their corresponding geographic/climatic variables, and the assigned hexaploid.

Abb.	Hexaploid percentage	Longitude (°E)	Latitude (°N)	Altitude (m)	Tmean (℃)	Annual precipitation (mm)	Hexaploid frequency group
HZ	6.30%	118.719	33.291	13	14.9	854	Low
GY	10%	119.348	32.864	5	15	946	frequency
PY	10.50%	116.301	29.214	13	17.8	1457	(0-50%)
LM	11%	118.182	34.103	21	14.7	732	
HH	13.80%	113.373	29.821	13	16.9	1262	
LG	19.80%	116.041	29.944	14	17.2	1405	
WS	23.75%	116.752	35.113	30	13.9	698	
TH	27.10%	120.183	31.257	4	15.7	1064	
DWS	37.50%	87.222	41.966	1053	10	82	
BST	53%	86.876	41.942	1052	10.4	80	sub-high
XH	56.70%	111.934	29.365	35	17.1	1275	frequency
DT	66%	112.693	28.811	28	17.5	1336	(50-85%)
TB	81.30%	115.828	29.965	13	17.2	1411	
TE	87%	84.116	42.919	2458	-3.3	256	high
CG	91%	124.284	45.27	127	5.1	431	frequency
JB	94%	128.911	43.854	370	3.2	580	(85-99%)
SB	95%	112.041	29.428	31	17.1	1264	
SH	98%	126.932	43.603	246	4.2	688	
FJ	98.30%	106.227	29.993	221	18.4	1056	
JLJ	98.30%	106.449	29.826	225	17.7	1085	
BM	98.30%	111.886	29.712	33	17	1226	
XS	98.84%	111.721	25.899	401	17.2	1456	
DC	100%	102.736	24.853	1889	16	1000	all-hexaploid
PA	100%	104.946	25.777	1610	14.8	1325	(100%)
LJ	100%	110.344	25.53	595	16.8	1586	
YC	100%	106.448	38.387	1119	8.8	206	
SMX	100%	111.154	34.782	345	13.9	492	
JY	100%	112.384	34.923	217	14	585	
XK	100%	132.264	45.228	68	4.4	573	
SF	100%	131.115	44.409	462	2.1	573	
R500	100%	87.505	43.758	1043	6.3	234	
IS	100%	87.747	47.393	501	5.6	147	
WLG	100%	87.123	47.234	479	6	131	
YL	100%	82.452	43.597	851	7.5	178	

Supplementary Table 3. Parameters of three geographic/climatic variables in 237 river or lake localities including the 34 sampled points that were used to predict geographic distribution pattern of hexaploids and tetraploids in *Carassius* species complex.

code	locality	altitude(m)	latitude(°N)	precipitation(mm)	hexaploid group index
1	Emuer river, Mohe county, Heilongjiang	491	52.89	456	298.189
2	Pangu river, Tahe county, Heilongjiang	430	52.674	466	244.009
3	Longjiang lake, Longjiang county, Heilongjiang	149	46.863	407	24.977
4	Huma river, Tahe town, Heilongjiang	370	52.343	479	189.618
5	Dongfanghong reservior, Hailun, Heilongjiang	247	47.646	567	55.624
6	Jielie river, Jiayin county, Heilongjiang	141	48.792	605	-44.13
7	Wuyuer river, Kedong county, Heilongjiang	164	45.061	613	-30.087
8	Wuli river, Fengman district, Jilin	273	43.728	692	34.364
9	Xinlicheng reservoir, Changchun, Jilin	211	43.623	640	-0.61
10	Songhua river, Qianguo county, Jilin	136	45.13	460	-4.098
11	Shihuiyao reservoir, Taonan county, Jilin	177	45.509	410	46.413
12	Jiaoliu river, Taonan county, Jilin	219	45.686	415	79.909
13	Xianghai reservoir, Tongyu county, Jilin	165	45.031	405	37.701
14	Hun river, Tonghua county, Jilin	482	41.67	899	140.405
15	Hailong reservoir, Meihekou county, Jilin	393	42.244	819	92.419
16	Xinkai river, Huanggu district, Liaoning	54	41.834	693	-149.675
17	Kou river, Xifeng county, Liaoning	208	42.728	713	-27.224
18	Qing river, Tieling city, Liaoning	94	42.573	671	-108.768
19	Longtun reservoir, Suizhong county, Liaoning	100	40.368	627	-91.103
20	Liangzi river, Changtu county, Liaoning	101	42.717	628	-88.993
21	Hulun lake, Xinyouqi county, Neimenggu	541	48.985	277	394.818
22	Dalinuoer lake, Keshiketengqi county, Neimenggu	1260	43.257	341	969.236
23	Wujia river, Wulateqianqi county, Neimenggu	1021	40.929	230	804.306
24	Daihai lake, Liangcheng county, Neimenggu	1220	40.555	384	920.23
25	Yuanhaizi lake, Wulantezhongqi county, Neimenggu	1023	41.247	188	819.712
26	Hongjianzhuo lake, Yijinhuoluoqi county, Neimenggu	1238	39.116	405	927.482
27	Chagannaori lake, Abagaqi county, Neimenggu	1015	43.425	254	793.279
28	Huangqihai, Chahaeryouyiqianqi county, Neimenggu	1265	40.835	358	966.278
29	Yellow river, Wuhai city, Neimenggu	1095	39.683	158	888.283
30	Qingshui river, Qingshuihe county, Neimenggu	1167	39.924	430	860.839
31	Beier lake, Xinyouqi county, Neimenggu	582	47.945	254	435.669
32	Xidayang reservoir, Tang county, Hebei	142	38.743	447	0.736
33	Luan river, Kuancheng county, Hebei	421	40.56	625	177.064
34	Gangnan reservoir, Pingshan count, Hebei	205	38.332	466	46.817

35	Douhe reservoir, Kaiping district of Tangshan, Hebei	43	39.745	636	-141.907
36	Nan lake, Yunhe district of Cangzhou, Hebei	10	38.299	582	-152.992
37	Yegoumen reservoir, Xingtai county, Hebei	470	37.198	585	228.472
38	Yongding river, Daxing district, Beijing	49	39.775	609	-128.194
39	Miyun reservoir, Miyun county, Beijing	146	40.461	530	-21.488
40	Ziyaxin river, Binhai new area, Tianjin	2	38.654	568	-154.907
41	Bao lake, Jinfeng district of Yinchuan, Ningxia	1111	38.447	197	888.211
42	Hequan lake, Yongning county, Ningxia	1111	38.311	203	886.187
43	Chenjia lake, Jinfeng district of Yinchuan, Ningxia	1109	38.507	195	887.23
44	Suyahu reservior, Yicheng district of Zhumadian, Henan	51	32.999	691	-157.547
45	Wei river, Weihui county, Henan	70	35.378	579	-104.036
46	Shishankou reservior, Luoshan county, Henan	75	32.011	1077	-262.52
47	Huai river, Huangchuan county, Henan	37	32.362	978	-262.056
48	Tuan river, Xinye county, Henan	92	32.602	818	-164.558
49	Luo river, Luoning county, Henan	364	34.356	590	136.628
50	Shimen reservoir, Huixian county, Henan	765	35.576	653	451.206
51	Wei river, Yangling district of Xianyang, Shanxi	437	34.232	619	188.015
52	Huama pool, Dingbian county, Shanxi	1313	37.685	302	1022.148
53	Fudi reservoir, Yinjun count, Shanxi	1085	35.394	577	742.114
54	Hongsiba reservoir, Nanzheng county, Shanxi	638	32.867	867	274.662
55	Xiewan reservoir, Luonan county, Shanxi	1001	34.033	727	622.915
56	Baliqiao reservoir, Hantai district of Hanzhong, Shanxi	526	33.107	876	178.632
57	Hanshui, Mian county, Shanxi	571	33.158	853	223.558
58	Yangjiawan reservoir, Jingbian county, Shanxi	1330	37.623	398	1005.355
59	Donglu river, Jingbian county, Shanxi	1402	37.491	405	1062.987
60	Wangyao reservoir, Ansai county, Shanxi	1279	36.903	467	940.164
61	Xiaosugan lake, Akesai county, Gansu	2811	39.065	37	2356.252
62	Dasugan lake, Akesai county, Gansu	2796	38.866	27	2346.842
63	Jiefangcun reservoir, Jinta county, Gansu	1301	39.935	81	1084.847
64	Kongdong reservoir, Kongdong district of Pingliang, Gansu	1542	35.544	543	1133.845
65	Black river, Linze county, Gansu	1413	39.283	109	1168.683
66	Yellow river, Qilihe district of Lanzhou, Gansu	1669	36.071	337	1306.327
67	Liujiaxia reservoir, Dongxiang autonomous County, Gansu	1744	35.842	424	1340.632
68	Jinchuan canyon, Yongchang county, Gansu	1913	38.308	211	1551.675
69	Liqiao reservoir, Shandan county, Gansu	2187	38.498	237	1771.674
70	Zhaling lake, Maduo county, Qinghai	4331	34.993	343	3521.107
71	Eling lake, Maduo county, Qinghai	4272	35.022	335	3474.556
72	Tuosuo lake, Delingha city, Qinghai	2818	37.156	149	2324.719

73	Keluke lake, Keluke town, Qinghai	2822	37.289	144	2329.752
74	Alake lake, Dulan county, Qinghai	4143	35.578	317	3373.274
75	Zhuonnai lake, Zhiduo county, Qinghai	4758	35.548	199	3923.544
76	Qinma lake, Zhiduo county, Qinghai	4918	35.589	174	4064.902
77	Sun lake, Zhiduo county, Qinghai	4882	35.928	162	4039.009
78	Gangnagemacuo lake, Maduo county, Qinghai	4198	34.326	406	3389.578
79	Rigecuochama lake, Maduo county, Qinghai	4210	34.327	411	3397.965
80	Hongyan reservoir, Wulumuqi county, Xinjiang	943	43.813	223	743.55
81	104tuan reservoir, Shayibake district of Wulumuqi, Xinjiang	903	43.787	225	709.568
82	Hongyanchi reservoir, Tianshan district of Wulumuqi, Xinjiang	1040	43.723	234	820.747
83	Santunbei reservoir, Tianshan district of Wulumuqi, Xinjiang	947	43.759	236	742.659
84	Kanasi lake, Habahe county, Xinjiang	1636	48.818	305	1297.823
85	Akekule lake, Ataile district, Xinjiang	2161	49.049	284	1742.067
86	Yixiekepati lake, Ruoqiang county, Xinjiang	3895	37.292	88	3241.595
87	Cheerchen river, Qiemo county, Xinjiang	2088	37.578	39	1752.337
88	Tekesi river, Tekesi county, Xinjiang	1067	43.228	208	851.273
89	Sailimu lake, Bole city, Xinjiang	2073	44.655	450	1612.319
90	Haizikou reservoir, Fuyun county, Xinjiang	1220	47.17	154	998.795
91	Wulungu river, Qinghe county, Xinjiang	1079	45.921	142	884.355
92	Daxihaizi reservoir, Weili county, Xinjiang	854	40.588	34	728.074
93	Yangzhuoyongcuo lake, Langkazi county, Tibet	4496	29.069	273	3677.058
94	Pumoyongcuo lake, Langkazi county, Tibet	5014	28.555	247	4116.574
95	Gerencuo lake, Shenzha county, Tibet	4651	31.102	280	3805.303
96	Angzicuo lake, Nima county, Tibet	4687	31.067	231	3851.046
97	Xurucuo lake, Angren county, Tibet	4716	30.342	220	3878.251
98	Jiezechaka lake, Ritu county, Tibet	4529	33.969	50	3779.69
99	Xianqiecuo lake, Ritu county, Tibet	4699	33.665	70	3914.653
100	Bumucuo, Geji county, Tibet	4576	33.259	96	3803.545
101	Taruocuo lake, Zhongba county, Tibet	4568	31.111	285	3734.561
102	Peikucuo, Jilong county, Tibet	4583	28.956	291	3743.656
103	Dajiacuo, Angren county, Tibet	5146	29.835	215	4237.706
104	Yuye lake, Shuanghu county, Tibet	4861	36.007	134	4030.586
105	Xiangyang lake, Anduo county, Tibet	4907	35.82	148	4064.268
106	erhai, Dali city, Yunnan	1960	25.842	1025	1320.228
107	Fuxian lake, Chengjiang county, Yunnan	1739	24.595	968	1153.64
108	Chenghai lake, Yongsheng county, Yunnan	1975	26.622	947	1358.371
109	Lugu lake, Yi autonomous county of Ninglang, Yunnan	2714	27.667	906	1987.871
110	Qilu lake, Tonghai county, Yunnan	1831	24.156	987	1223.859
111	Xingyun lake, Jiangchuan county, Yunnan	1845	24.364	977	1238.883
112	Yangzonghai lake, Yiliang county, Yunnan	1824	24.902	1004	1213.062

113	Wanfeng lake, Yixing county, Guizhou	833	24.761	1197	325.317
114	Gezhai reservoir, Xixiu district of Anshun, Guizhou	1272	26.182	1284	663.958
115	Jianghong reservoir, Xixiu district of Anshun, Guizhou	1331	26.157	1302	707.292
116	Loujia lake, Xixiu district of Anshun, Guizhou	1449	26.25	1313	802.107
117	Yaoshang reservoir, Zhongshan district of Liupanshui, Guizhou	1876	26.588	1081	1232.732
118	South panjiang, Ceheng county, Guizhou	791	24.738	1231	279.368
119	Caohai lake, Yi autonomous county of Weining, Guizhou	2183	26.844	936	1535.328
120	Pingyan river, Libo county, Guizhou	676	25.185	1407	127.205
121	Qionghai lake, Xichang county, Sichuan	1518	27.825	995	963.053
122	Ma lake, Leibo county, Sichuan	1286	28.408	956	782.752
123	Jinsha river, Suijiang county, Sichuan	1055	28.526	1005	574.631
124	Zhaoyang lake, Pujiang county, Sichuan	629	30.131	1531	51.494
125	Modi river, Jinniu district of Chengdu, Sichuan	518	30.708	973	139.1
126	Shiyang river, Tianquan county, Sichuan	853	30.011	1478	255.07
127	Yangtze river, Wanzhou district, Chongqing	194	30.652	1222	-211.008
128	Damotan, Pengshui county, Chongqing	255	29.698	1075	-113.511
129	Jialingjiang, Beibei district, Chongqing	183	29.921	1089	-177.843
130	Chongluo lake, Hezhou city, Guangxi	489	24.291	1500	-59.121
131	Bagui river, Youjiang district of Baise, Guangxi	435	24.019	1143	10.666
132	Chengbi river, Lingyun county, Guangxi	706	24.266	1227	209.529
133	Hongshui river, Tian'e county, Guangxi	633	25.162	1221	151.262
134	Xinfengjiang reservoir, Dingyuan county, Guangdong	100	23.767	1919	-518.433
135	Xi river, Xinhui district of Jiangmen, Guangdong	0	22.416	1540	-480.615
136	Xunwushui, Longchuan county, Guangdong	198	24.443	1641	-346.822
137	Dongjiang river, Boluo county, Guangdong	22	23.274	1941	-590.826
138	Huangsha reservoir, Huiyang district of Huizhou, Guangdong	123	22.984	2047	-541.023
139	Han river, Xiangqiao district of Chaozhou, Guangdong	14	23.668	1492	-452.644
140	Rongjiang river, Chaoyang district of Shantou, Guangdong	7	23.436	1525	-469.259
141	Xiangjiang river, Yanfeng district of Hengyang, Hunan	63	26.863	1411	-383.569
142	Pingshui river, Miao autonomous county of Chengbu, Hunan	650	26.247	1498	76.968
143	Sixiang river, Dong autonomous county of Tongdao, Hunan	413	26.278	1365	-77.606
144	Mishui, You county, Hunan	87	26.975	1543	-406.005
145	Dongjiang lake, Zixing county, Hunan	340	25.894	1511	-185.688

146	Yuanshui, Yuanling county, Hunan	153	28.272	1395	-302.488
147	Jiulongjiang, Longwen district of Zhangzhou, Fujian	56	24.55	1428	-396.449
148	Minjiang, Yanping district of Nanping, Fujian	137	26.556	1605	-384.604
149	Xi lake, Gulou ddistrict of Fuzhou, Fujian	15	26.093	1367	-409.91
150	Jinshui lake, Minhou county, Fujian	147	26.15	1437	-322.455
151	Yahu reservoir, Zhaoan county, Fujian	111	23.817	1346	-324.73
152	Junshan lake, Jinxian county, Jiangxi	19	28.483	1330	-393.036
153	Qingshan lake, Qingshanhu district of Nanchang, Jiangxi	19	28.7	730	-199.688
154	Ganjiang, Nanchang county, Jiangxi	22	28.303	1196	-347.512
155	Yangtze river, Jiujiang county, Jiangxi	16	29.746	1447	-432.349
156	Wojiang river, Longnan county, Jiangxi	219	24.879	1654	-333.218
157	Yuanshui, Yuanzhou district of Yichun, Jiangxi	89	27.799	1563	-410.218
158	Dongjiang, Nancheng county, Jiangxi	156	27.604	1687	-394.468
159	Yangtze river, Zhijiang county, Hubei	37	30.356	1121	-309.469
160	Malan river, Fang county, Hubei	468	32.059	871	131.214
161	Yangtze, Shishou county, Hubei	33	29.767	1211	-342.182
162	Zhang river, Dangyang county, Hubei	85	30.965	1000	-230.108
163	Liangzi lake, Jiangxia district of Wuhan, Hubei	20	30.204	1318	-387.167
164	Huama lake, E'cheng district of E'zhou, Hubei	21	30.261	1382	-406.903
165	Chao lake, Lujiang county, Anhui	9	31.484	1080	-318.822
166	Baitu lake, Zongyang county, Anhui	7	30.886	1324	-399.464
167	Nan lake, Xuanzhou district of Xuancheng, Anhui	8	31.085	1202	-359.211
168	Wabu lake, Shou county, Anhui	20	32.314	861	-238.576
169	Huai river, Huoqiu county, Anhui	20	32.447	835	-230.114
170	Quanriver, Linquan county, Anhui	33	33.01	862	-227.595
171	Ying river, Yingquan district of Fuyang, Anhui	29	32.922	878	-236.139
172	Guo river, Qiaocheng district of Bozhou, Anhui	38	33.838	792	-200.326
173	Changdang lake, Jintan county, Jiangsu	2	31.598	1101	-331.338
174	Yangtze river, Jianye district of Nanjing, Jiangsu	7	32.052	1002	-294.986
175	Xichao river, Tinghu district of Yancheng, Jiangsu	1	33.553	989	-294.775
176	Shaoxian river, Guangling district of Yangzhou, Jiangsu	7	32.454	1004	-295.356
177	Yangcheng lake, Kunshan county, Jiangsu	4	31.404	1056	-315.314
178	Shilianghe reservoir, Donghai county, Jiangsu	21	34.752	881	-242.523
179	Dingshan lake, Qingpu district, Shanghai	4	31.12	984	-292.323
180	Feiyun river, Rui'an county, Zhejiang	5	27.793	1653	-509.174
181	Xinan river, Jiande county, Zhejiang	60	29.498	1469	-402.95
182	Qiantang river, Xiaoshan district of Hangzhou, Zhejiang	7	30.375	1234	-370.832
183	Xinan lake, Nanhu district of Jiaxing, Zhejiang	7	30.747	1091	-324.532
184	Fenghuajiang, Fenghua county, Zhejiang	17	29.649	1405	-418.058
185	West lake, Xihu district of Hangzhou, Zhejiang	15	30.255	1369	-407.719

186	Xiuling reservoir, Huangyan district of Taizhou, Zhejiang	13	28.528	1565	-473.673
187	Yellow river, Zhangqiu county, Shandong	17	37.071	562	-141.558
188	Dongping lake, Dongping county, Shandong	40	35.929	619	-141.53
189	Dushan lake, Weishan county, Shandong	28	35.057	701	-178.524
190	Nanshasu river, Lanshan district of Linyi, Shandong	74	35.079	836	-183.661
191	Hehua lake, Wulu county, Shandong	7	37.739	577	-154.263
192	Yongfeng river, Kenli county, Shandong	3	37.53	589	-161.601
193	Yi river, Hedong district of Linyi, Shandong	59	34.958	846	-199.459
194	Qingfeng lake, Dongying district of Dongying, Shandong	1	37.412	590	-163.669
195	Zhaoyang lake, Yutai county, Shandong	30	34.972	712	-180.458
196	Yu river, Nanjiao district of Datong, Shanxi	1236	40.241	371	937.53
197	Hui river, Shuocheng district of Shuozhou, Shanxi	1088	39.293	436	792.671
198	Yellow river, Liulin county, Shanxi	760	37.44	489	501.119
199	Fen river, Qingxu county, Shanxi	761	37.541	441	517.476
200	Anyi lake, Yanhu district of Yuncheng, Shanxi	359	35.053	522	154.834
201	Zhangze reservoir, Tunliu county, Shanxi	909	36.274	595	590.31
202	Wuxing lake, Yongji county, Shanxi	347	34.886	513	147.622
203	Yellow river, Hequ county, Shanxi	938	39.153	445	664.727
204	Hongze Lake, Sihong county, Jiangsu	13	33.291	854	-241.488
205	Gaoyou Lake, Gaoyou county, Jiangsu	5	32.864	946	-278.067
206	Poyang Lake, Duchang county, Jiangxi	13	29.124	1457	-438.492
207	Luoma Lake, Suyu district of Suqian, Jiangsu	21	34.103	732	-194.987
208	Honghu Lake, Honghu county, Hubei	13	29.821	1262	-375.227
209	Longgan Lake, Huangmei county, Hubei	14	29.944	1405	-420.356
210	Weishan Lake, Weishan county, Shandong	30	35.113	698	-175.854
211	Taihu Lake, Wuxi city, Jiangsu	4	31.257	1064	-317.99
212	Dawusong Lake, Heshuo county, Xinjiang	1053	41.966	82	879.324
213	Bositeng Lake, Heshuo county, Xinjiang	1052	41.942	80	879.119
214	Xihu Lake, Jinshi county, Hunan	35	29.365	1275	-361.397
215	Dongting Lake, Xiangyin county, Hunan	28	28.811	1336	-387.248
216	Taibai Lake, Huangmei county, Hubei	13	29.965	1411	-423.107
217	Tian’e Lake, Hejing county, Xinjiang	2458	42.919	256	1994.31
218	Chagan Lake, Qianguo county, Jilin	127	45.27	431	-2.162
219	Jingbo Lake, Ningan county, Heilongjiang	370	43.854	580	151.315
220	Shanbo Lake, Anxiang county, Hunan	31	29.428	1264	-361.145
221	Songhua Lake, Jiaohe city, Jilin	246	43.603	688	13.076
222	Fujiang, Hechuan district, Chongqing	221	29.993	1056	-135.514
223	Jialingjiang, Beibei district, Chongqing	225	29.826	1085	-141.633
224	Beimin Lake, Jinshi county, Hunan	33	29.712	1226	-347.049
225	Xiaoshui, Shuangpai county, Hunan	401	25.899	1456	-117.162

226	Dianchi, Chenggong district of Kunming, Yunnan	1889	24.853	1000	1268.462
227	Puan, Puan county, Guizhou	1645	25.538	1340	956.196
228	Lijiang, Xing'an county, Guangxi	595	25.530	1586	2.329
229	Yellow river, Xingqing district of Yinchuan, Ningxia	1119	38.387	206	891.937
230	Yellow river, Hubin district of Sanmenxia, Henan	345	34.782	492	152.648
231	Yellow river, Jiyuan city, Henan	217	34.923	585	16.174
232	Xingkai Lake, Mishan city, Heilongjiang	68	45.228	573	-97.062
233	Suifen River, Suifenhe city, Heilongjiang	462	44.409	573	230.583
234	500 reservoir, Fukang city, Xinjiang	503	44.18	177	392.092
235	IrtySh River, Aletai district, Xinjiang	501	47.393	147	402.274
236	Wulungu Lake, Aletai district, Xinjiang	479	47.234	131	388.991
237	Yili River, Gongliu county, Xinjiang	851	43.597	178	681.257

Supplementary Table 4. Nucleotide diversity of *transferrin* alleles between hexaploids and tetraploids in the 34 sampled populations.

Abbreviation of population	Nucleotide diversity of transferrin (π±SD)	
	Tetraploids	Hexaploids
HZ	0.0443±0.0213	0.0678±0.0339
GY	0.0432±0.0208	0.0511±0.0261
PY	0.0240±0.0117	0.0851±0.0431
LM	0.0082±0.0042	0.0135±0.0073
HH	0.0060±0.0032	0.0644±0.0324
LG	0.0407±0.0197	0.0987±0.0484
WS	0.0423±0.0205	0.0521±0.0257
TH	0.0864±0.0413	0.0526±0.0255
DWS	0.0402±0.0195	0.0469±0.0229
BST	0.0415±0.0201	0.0498±0.0240
XH	0.0154±0.0079	0.0903±0.0435
DT	0.0335±0.0164	0.1110±0.0531
TB	0.0584±0.0290	0.0913±0.0436
TE	0.0483±0.0242	0.0487±0.0234
CG	0.0519±0.0264	0.0448±0.0216
JB	0.0519±0.0277	0.0704±0.0337
SB	-	0.0859±0.0412
SH	-	0.0536±0.0257
FJ	-	0.0679±0.0326
JLJ	-	0.0720±0.0346
BM	-	0.0646±0.0310
XS	-	0.0591±0.0283
DC	-	0.0507±0.0244
PA	-	0.0523±0.0251
LJ	-	0.0475±0.0228
YC	-	0.0461±0.0221
SMX	-	0.0512±0.0246
JY	-	0.0527±0.0253
XK	-	0.0533±0.0256
SF	-	0.0488±0.0235
R500	-	0.0464±0.0223
IS	-	0.0434±0.0209
WLG	-	0.0394±0.0191
YL	-	0.0430±0.0208
